# Studying Ice with Environmental Scanning Electron Microscopy

**DOI:** 10.3390/molecules27010258

**Published:** 2021-12-31

**Authors:** Elzbieta Pach, Albert Verdaguer

**Affiliations:** Institut de Ciència de Materials de Barcelona (ICMAB-CSIC), Campus de la UAB, E-08193 Bellaterra, Spain; averdaguer@icmab.es

**Keywords:** water, ice, scanning electron microscopy, ambient conditions, nucleation

## Abstract

Scanning electron microscopy (SEM) is a powerful imaging technique able to obtain astonishing images of the micro- and the nano-world. Unfortunately, the technique has been limited to vacuum conditions for many years. In the last decades, the ability to introduce water vapor into the SEM chamber and still collect the electrons by the detector, combined with the temperature control of the sample, has enabled the study of ice at nanoscale. Astounding images of hexagonal ice crystals suddenly became real. Since these first images were produced, several studies have been focusing their interest on using SEM to study ice nucleation, morphology, thaw, etc. In this paper, we want to review the different investigations devoted to this goal that have been conducted in recent years in the literature and the kind of information, beyond images, that was obtained. We focus our attention on studies trying to clarify the mechanisms of ice nucleation and those devoted to the study of ice dynamics. We also discuss these findings to elucidate the present and future of SEM applied to this field.

## 1. Introduction

Ice nucleation and water freezing are naturally occurring processes on Earth with an enormous impact on climate, geology, and life. Understanding the mechanisms governing these processes is essential for predicting the future of our planet. Without knowing more about ice formation, it is impossible to build robust snow or ice-cloud theories for atmospheric and climate models or to extrapolate laboratory studies to environmental conditions with confidence. A few years ago, the ten questions that science needs to examine to gain this knowledge were established [1]. The first question raised was to understand how ice nucleation (IN) occurs in the troposphere.

The formation of ice in the troposphere, both on the Earth’s surface and in clouds, usually takes place on solid surfaces through heterogeneous nucleation. Many factors can affect heterogeneous nucleation and not all surfaces induce ice nucleation with the same efficiency. The properties of surfaces at the nanometer range play a key role in heterogeneous nucleation due to the size of the initial ice nuclei, which is in the order of few to tens of nanometers. To understand how that happens, the molecular bases of the interaction of water molecules with surfaces need to be studied. Many different techniques can be used to study water interaction with surfaces and heterogeneous nucleation, such as photon spectroscopy [2], optical microscope [3,4], and others. Nevertheless, all these techniques lack the spatial resolution needed to study it at the required nanometer range. Important knowledge has been obtained with the use of scanning probe microscopy techniques, which allowed for the study of ice on surfaces with high spatial resolution in different environmental conditions [5,6]. Unfortunately, such techniques always pay the price of the undesirable interaction between the probe and the water layers, perturbing the measurements [7].

In recent years, with the rapid advancement in instrumentation as well as simulation capabilities, important advances in the process of understanding of ice nucleation and ice dynamics emerged [3,8,9,10,11,12]. One of the main techniques applied today in the investigation on the topic is environmental scanning electron microscopy, ESEM. In a recently published review on the “Applications of ESEM on Materials Science: Recent Updates and Look Forward” [13], the authors dedicated one part of the in situ environmental scanning electron microscopy operations to the observations of phase transitions of water. Although the review is very extended and complete on the technique in question and its use in Materials Sciences in general, it referenced limited published works relating to ice. In this paper, we will review the use of the ESEM technique in the investigation concerning the ice nucleation already found in the literature and propose new possibilities this technique can offer for future investigations on this subject.

## 2. Results

### 2.1. Environmental SEM and the Study of Ice

**Figure 1:**
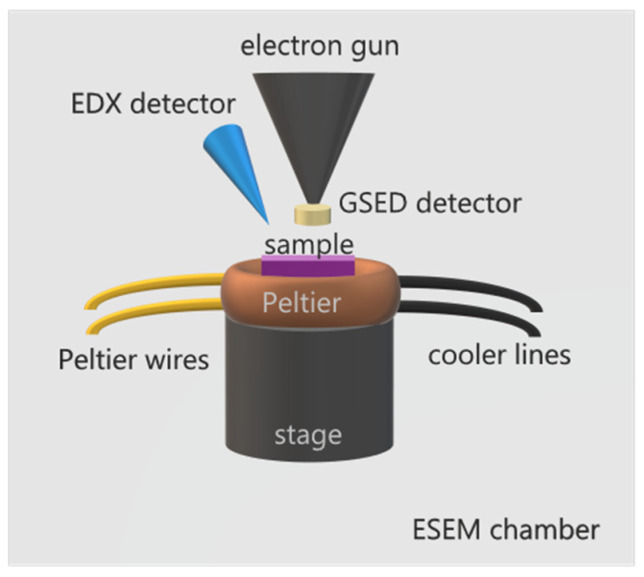
Scheme of ESEM for ice formation and sublimation studies. The sample is placed on top of a Peltier element, able to cool down a sample to temperatures around −30 °C, equipped with cooler circuit lines and Peltier wires for temperature control. The Peltier stage is assembled on top of SEM’s motorized stage. A specially designed GSED detector is attached to the gun nozzle. Additionally, an EDX detector may be used for spectroscopic analysis. The assembly is enclosed in the ESEM chamber, which may be filled with water vapor up to 100 % RH or even supersaturation conditions.

Environmental scanning electron microscopy (ESEM) was developed to overcome the limitations of the classical SEM, operated at high vacuum condition, required for the operation of the electron gun. This condition limited the range of specimens compatible with the technique to conductive, dry, and stable samples. The development of differentially pumped diaphragms allowed the introduction of a gas medium in the specimen chamber while maintaining high vacuum conditions for the electron gun [14,15,16]. This, along with the development of a specific gaseous secondary electron detector (GSED), opened the way to study insulated and wet specimens with the pressure in the chamber reaching up to 50 Torr (~6000 Pa) [17]. The maximum pressure limit depends on the instrument and varies from one model to another. The main reason to limit the gas pressure in the chamber is to protect the electron gun, which is sensitive to vacuum levels. High vacuum conditions must be met to avoid electrical discharge or arcing. In ESEM standard operation conditions, the gas introduced into the chamber can be water vapor, which greatly facilitates the investigations of water/solid interfaces, heterogeneous ice nucleation, and ice dynamics. To study ice, the sample temperature may also be controlled using a cooling stage, usually a Peltier element. Thus, by varying the water partial pressure and using a Peltier holder to control the temperature, we can obtain environmental conditions with the temperatures reaching down to around −30 °C and relative humidity conditions (RH) reaching up to 100% or even supersaturation conditions. Moreover, with the energy dispersive X-ray detector, often attached to the ESEM, it is also possible to determine the chemical elements and their relative abundance in the specimen under study, it being a surface or small particle. These technical characteristics are a great advantage of ESEM over other techniques, such as AFM or optical microscopy, especially when working with natural materials of an unknown surface composition, as it often happens in studies related to cloud physics. In heterogeneous nucleation experiments combining ESEM with EDX analysis, without the need of coating the insulating samples to compensate for charge formation, one can determine the composition of the sites on the specimen where ice nucleation occurs. A scheme of a typical set up for ESEM studies on ice formation and sublimation with its main elements is displayed on Figure 1.

The first commercial ESEM was presented in 1980 by the Electroscan Corporation, but, soon after, other companies followed to introduce the ESEM capabilities into their instruments. Nowadays, ESEM chambers are available from different manufactures and are widely used in investigation in many areas of science, such as materials sciences, biology, or nanotechnology. Before ESEM, ice nucleation investigation was based on diverse techniques developed for that purpose including single particle methods, such as rapid expansion cloud chamber [18], mixing chamber [19], continuous flow diffusion chamber [20,21], and slow expansion cloud chamber to simulate realistic updrafts [22], or immersion freezing methods, such as freezing assays [23], microfluidic-flow based methods [24,25,26,27], and differential scanning calorimeter [28,29,30,31,32,33,34]. All of the single particle methods are based on exposing the aerosol particles to a cold supersaturation condition and counting the ice crystals that nucleate and grow to a detectable size. Light microscopy has generated highly detailed images of ice crystals [35,36], but a combination of working distance constraints, diffraction limits to resolution, and transmitted light illumination have prevented visible imaging of mesoscopic surface features. With the ESEM development came the possibility of observing water-vapor-particle interactions in situ and at a sub-micrometer range. However, there is a concern regarding the use of electron-based techniques for the study of water-surface interactions, because of a possible dissociation of water molecules by electron beam [37]. The interference of the electron beam with water molecules or the ice itself may affect the process of the ice growth.

The technique is continuously evolving and, as a recent example, a new system combining ESEM capabilities with an X-ray spectroscopy analytical platform was developed to directly observe the evolution of organic particles with increasing RH [38]. The new setup allowed for the probing of the chemical state, morphology, and functional groups of individual particles with two combined techniques: computer-controlled scanning electron microscopy with energy dispersive X-ray spectroscopy (CCSEM\EDX) and scanning transmission X-ray microscopy with near-edge X-ray absorption fine structure spectroscopy (STXM\NEXAFS).

### 2.2. Heterogeneous Ice Nucleation

**Figure 2:**
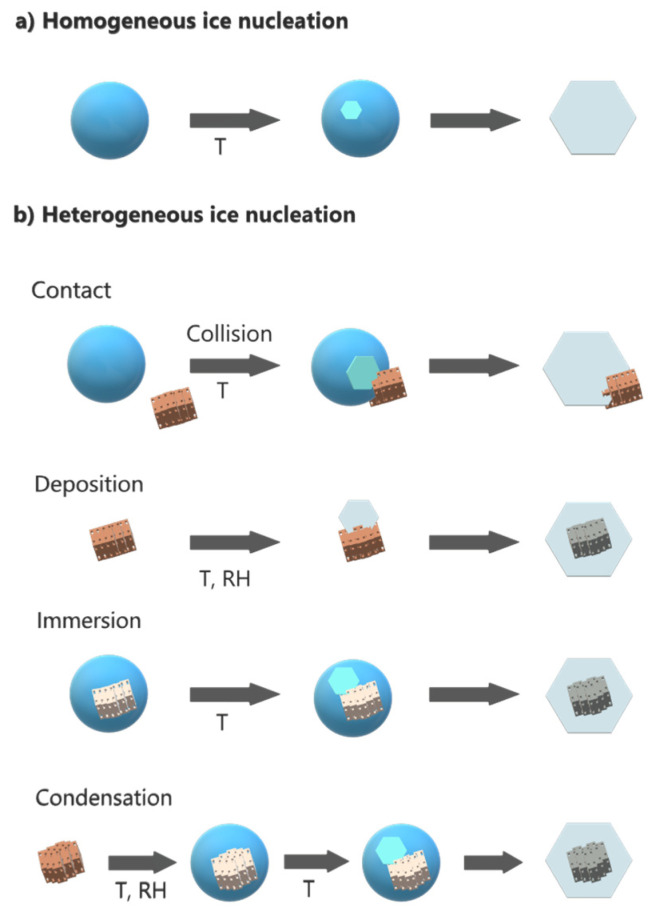
(**a**) Homogeneous and (**b**) heterogeneous mechanisms of ice formation in the troposphere.

Different mechanisms of heterogeneous ice formation in the troposphere have been proposed (Figure 2): contact freezing (an ice nucleating particle triggers freezing by contact with a supercooled droplet), deposition freezing (water vapor condensation on a particle initiates ice formation), immersion freezing (a particle immersed in a droplet triggers freezing), and condensation freezing (water condensation on a particle initiates freezing) [39,40]. 

**Figure 3:**
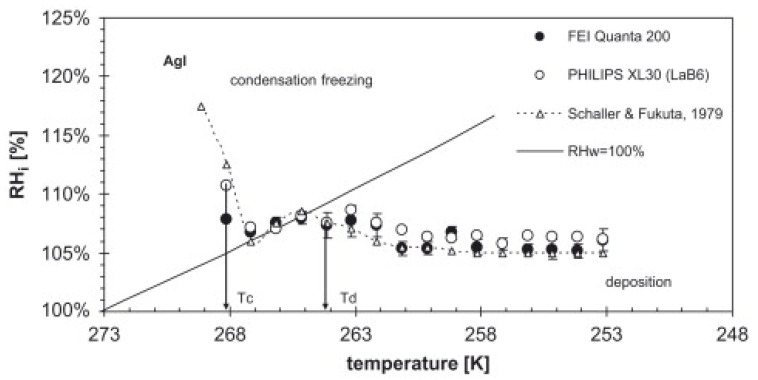
Supersaturation vs. temperature diagram for silver iodide.

In 2007, Frank Zimmermann et al. [41] presented the first article, to our knowledge, on the use of ESEM for the study of heterogeneous ice nucleation. The authors studied the heterogeneous IN on individual atmospheric aerosol particles (synthetic silver iodide, natural kaolinite, and montmorillonite particles) at temperatures between 250 K and 270 K. It was shown that ice formation could be observed in situ by increasing the water pressure in the sample chamber at a constant temperature until water supersaturation is reached. Using this setup, by varying the water vapor pressure and the temperature of the sample, two different heterogeneous nucleation modes can be investigated: deposition nucleation and condensation freezing nucleation [42] (see Figure 3). In the deposition mode, ice is formed on the surface from an ice supersaturated vapor environment (i.e., ice relative humidity is >100%, but water relative humidity, RHw < 100%). In condensation freezing, water adsorbs on the surface first, and then heterogeneous ice nucleation takes place (RHw > 100%). The authors used EDX for chemical detection of the nucleation sites. Although a lateral resolution of 10–20 nm was observed on atmospheric aerosol particles in ice nucleation experiments, the magnification was limited to avoid the melting of the ice crystals. Thus, the smallest features that could be detected reliably were on the order of 200 nm and it was not possible to visualize the formation of the ice nucleus itself. Instead, ice crystal growth after nucleation was witnessed. Nevertheless, it was enough to determine the particle responsible of each nucleation. An important outcome of this study was that the environmental conditions (temperature and water vapor pressure) at which ice nucleation happened depended strongly on the nature of the mineral used in the experiments.

More evidence on the dependence on mineralogy was presented by the same authors the following year. Using the same technique, they examined ice nucleation on nine pure minerals present in mixed-phase clouds [43]. The authors observed that, for deposition nucleation, RHi at which ice nucleation took place showed a strong increase with decreasing T for kaolinite, montmorillonite, and hematite, while for illite, albite, quartz, and calcite, RHi would remain constant. This behavior is probably strongly related to the different structures of the water molecules at the interface with the minerals. In this paper, we want to point out that water adsorption on surfaces already occurs at RHw < 10% in the form of thin films [44], with water molecules adopting structures that can be very different from bulk liquid water depending on the solid surface [45,46,47]. However, this effect could also be due to the prevalence of certain ice nucleating sites with the right combination of size and shape of pores/cracks/steps that either ease or difficult ice formation in the different mineralogy, as suggested by the pore condensation and freezing theory [48].

A novel ice nucleation ESEM experimental platform with a cryogenic temperature controller was presented in 2016 by Wang et al. [49]. The aim of this system was to obtain wider ranges of cooling rates, lower temperatures, and RH as well as having a more precise control over these parameters to experimentally reproduce the extreme conditions in mixed-phase clouds. The ESEM platform included a sample holder, a cryo-stage with cooling and heating capabilities (liquid nitrogen and resistive heating), and a base attached to the microscope’s motorized stage. The lateral resolution of the images was around 10 nm. For a proof-of-concept study in isothermal ice nucleation experiments, the authors selected layered kaolinite platelets and discovered that the ice formation occurs preferentially at the edges of those platelets, rather than on their basal planes. Trying to elucidate the reasons behind this behavior, the authors performed additional chemical analyses of the particles by the EDX and post-experimental STXM/NEXAFS. The chemical differences between the edges and the basal planes, specifically the presence of OH groups on the edges, were pointed out as the reason for being potential ice nucleating sites.

**Figure 4:**
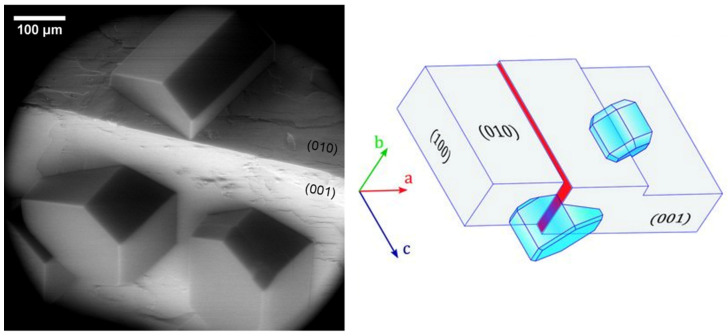
(**Left**) Preferential orientation of ice crystals nucleated on the surface of weathered feldspar on (010) and (001) surface at 241 K. (**Right**) Schematic drawing of ice crystals nucleating from their (01$\bar{1}$0) planes on the steps with (100) orientation (red filled surface and the hidden face of the step on the (010) face of feldspar). Adapted with permission from [8]. Copyright 2021 American Association for the Advancement of Science.

A breakthrough in the understanding of the nature of ice growth on mineral samples came in 2017, when Kiselev et al. [8] directly observed the formation of hexagonal ice crystals on K-feldspar during electron microscopy experiments. K-feldspar mineral particles are believed to be IN active components of mineral dust present in natural clouds, responsible for many of the nucleation events in the atmosphere [50,51]. Ice crystals were found to grow at or in the vicinity of surface defects, such as steps, cracks, or pores, but it was also found that the crystals align with the basal plane to the (100) crystallographic face of the mineral (Figure 4), indicating a template imposed by the substrate. Additionally, their molecular-scale computer simulations indicated that this alignment arises from the preferential nucleation of prismatic crystal planes of ice on high-energy (100) surface of feldspar. The authors claimed that microscopic patches of the (100) face were exposed at surface defects, making them very efficient at inducing ice-nucleation. Previous experimental and modeling studies already pointed at several factors being responsible for high IN efficiency of particles. The most important were the crystal lattice match [52,53,54,55], presence of surface hydroxyl groups [56,57,58,59,60], and surface defects that locally enhance the density of adsorbed water molecules [48,61,62] or reduce their surface diffusivity [63]. The importance of defects was demonstrated before the cited article in experiments where substrates with excellent lattice match to ice were found to be even more effective in ice nucleating through the introduction of surface defects [64]. It is worth noting here that, to this day, the importance of the lattice match is not clear, and some molecular-scale simulations even question its implication [65,66]. The same happens with the role of the surface hydroxyl groups, which is believed to be of a complex nature [57,58,59,60,65,67,68,69]. Nevertheless, Kiselev et al., based on the atomistic computer simulations, drew the conclusion that it is indeed the presence of the hydroxyl groups on the surface of the (100) patches that plays an important role in the nucleation by allowing ice-like structures to form in the cases where there is no apparent epitaxial match. Hence, deeper investigation into the role of the OH groups in ice nucleation efficiency on dust particles and surfaces is needed.

The ESEM higher resolution capabilities were also key in exposing the microscopic step by step pathway of ice crystals’ formation in a supersaturated water vapor environment where two different origins of the steps were unveiled [70]. The hexagonal ice crystals were found either emerging from the screw dislocations or from the initial steps. The authors analyzed ice crystal growth rate at temperatures between −12 and −22 °C with the fastest growth rate found at −14 °C. According to the authors, this behavior is due to the formation of a quasi-liquid-layer on the surface of the crystal, a consequence of pre-melting. At temperatures higher than −12 °C, the pre-melting layer covers the entire ice crystal smoothly. This prevents adsorption of the water molecules from the vapor on the ice crystal and its grow. At low temperatures, the very low pre-melting of the ice surface makes it very smooth, which slows down the growth of the ice. It has been shown by large-scale computer simulations that, in the range of about 80 K below the melting point, the main facets of ice may exist in up to three different surface phases with distinct degrees of surface disorder [12]. The authors observed pre-melting mediated surface smoothening accompanied by an increase of step free energies.

Ice nucleation on porous particles can be described by the pore condensation and freezing theory, which was revisited and updated in 2019, based on the latest experimental results [71]. In those materials, the confinement of water in the pores acts as an IN promoter. The effect of pores was clearly observed in K-feldspar minerals after the first indications by Kiselev et al. in our ESEM experimental study, showing how ice nucleation and freezing is dominated by pores for these minerals [72].

**Figure 5:**
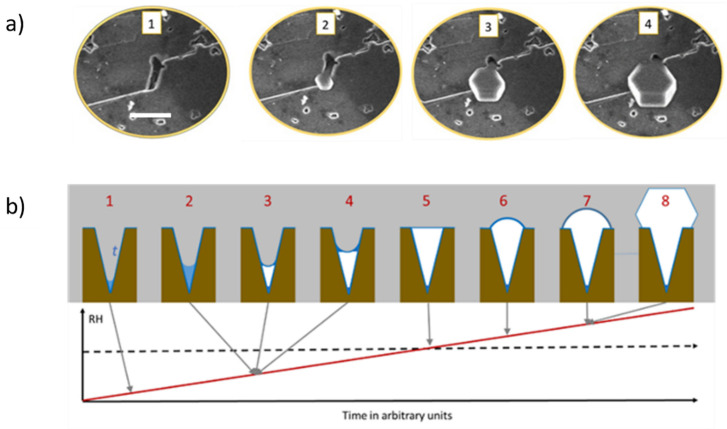
(**a**) Experimental ESEM images of the formation of ice within a pore and emerging out of the pore (adapted with permission from [72]. Copyright 2019 American Chemical Society). (**b**) Illustration of the theory of pore condensation and freezing from conical or wedge-shaped pore. The scale bar in (**a**) is 5 µm.

**Figure 6:**
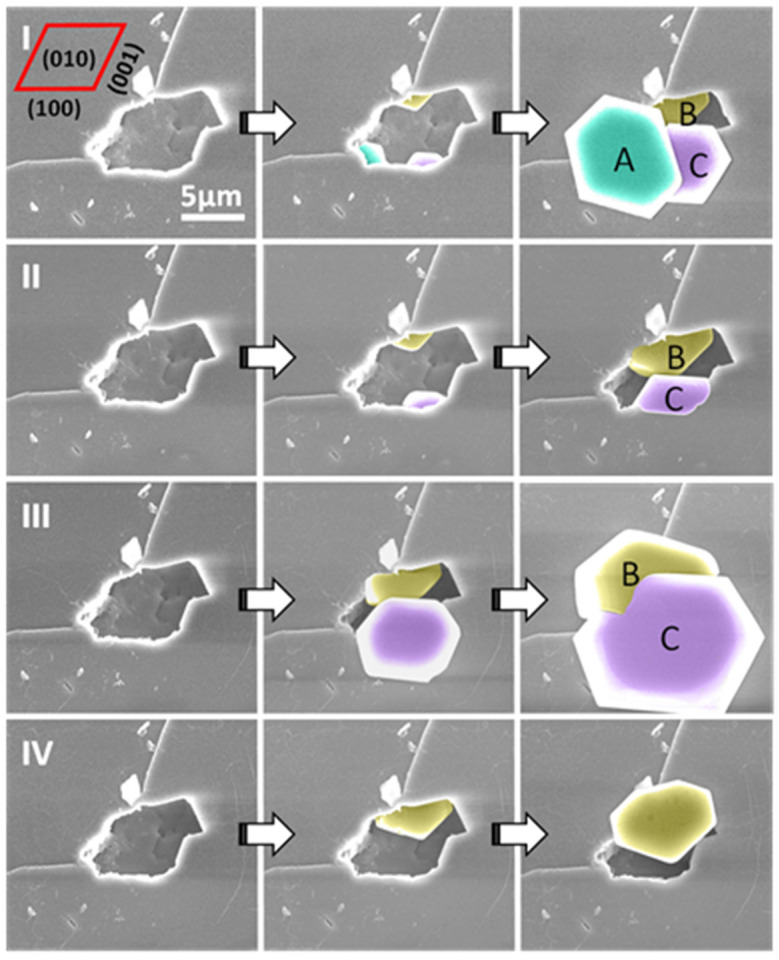
ESEM images of four nucleation cycles (**I**–**IV**) from a single pore on the (010) face of the feldspar crystal. The crystal grown from the exact site inside the pore always shows the same orientation; however, crystals grown from different sites inside the pore can show different orientations: A, B and C show slightly different orientations, but B and C, which appear in cycles (**I**–**III**), always show the same orientation. Figure adapted with permission from [72]. Copyright 2019 American Chemical Society.

In our study, we were able to observe, in situ and in real-time, the filling of the pore and the emerging out of it in the form of a hexagonal ice crystal (Figure 5a). Consecutive cycles of ice formation in the same pore would result in the same crystal orientation and form, indicating, as observed before by Kiselev et al., an over imposed template from the surface to the ice crystallographic growth (Figure 6). These results, offering a direct observation by ESEM imaging of the filling of the pores with ice and then the growth of the hexagonal ice crystals out of the pores, were described in more detail by theoretical work, in a step-by-step explanation of the process, by Macrolli et al. in 2020 [73]. In a conical or wedge-shaped pore at low RH, a water layer forms on the surface of the pore and a small amount of water condensates on the bottom. It remains liquid until reaching the volume of critical ice embryo, when ice starts to grow from the vapor phase. With a further increase of RH, the ice fills more and more of the accommodating pore until the formation of a cap on its top. Only when the angle of the cap reaches the critical value, the ice growth becomes unrestricted yielding the typical hexagonal ice crystal growth (Figure 5b).

Carbon-based particles found in the troposphere account for up to 50% of all the particles, many of them being of graphitic-like structure, called soot particles. For this reason, carbon nanomaterials are studied as ice nucleation promoters, especially graphene and its derivatives due to their intrinsic and functionalized surface properties [46,68,74,75]. For example, the sublattice of graphite nearly matches that of the natural ice, which could favor the epitaxial growth of the stable hexagonal ice. Graphene oxide (GO), due to the presence of many hydrophilic functional groups, can capture water molecules and, in this way, it may favor ice formation. Recently, synthesized porous composite of 3D reduced graphene oxide (rGO) and silica dioxide nanoparticles [76] with narrow-sized SiO_2_ nanoparticles uniformly distributed across rGO structure was tested as an ice nucleating promotor. It was found that the composite promotes hexagonal ice growth via a lattice match between the ice and its structure. The in situ ESEM ice growth observation confirmed the enhanced ice nucleation performance by ice crystal formation events starting from −8 °C and 5–8% RH. In addition, it was observed that ice nucleation would start in large cavities in the rougher regions of the composite, in line with other, previously mentioned studies.

**Figure 7:**
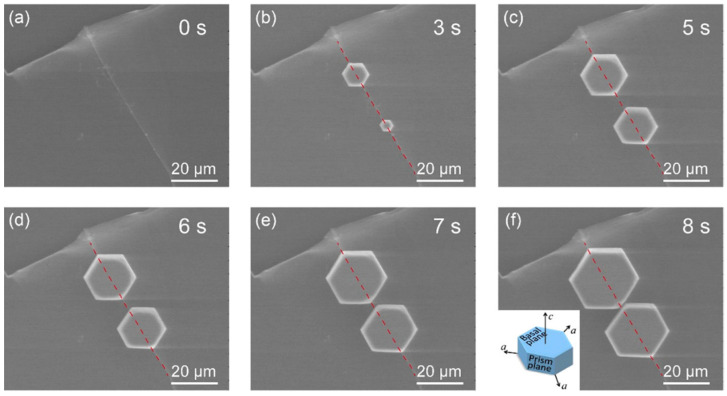
In situ ESEM image series for the atomic-step-induced aligned growth of hexagonal ice crystals on the HOPG surface at −14 °C. (**a**) Morphology of the atomic step on the HOPG surface. (**b**–**f**) Nucleation and growth dynamics of hexagonal crystals in the step area, with their (0001) basal plane parallel to the HOPG surface. The crystals were further aligned by the atomic step, as one of their a-axes coincides with the step edge during their growth. The inset in (**f**) shows the basic lattice unit of the hexagonal ice crystal with two basal planes (0001) and six equivalent prism planes (1010). The crystal has three identical a-axes. The red dotted line in each image indicates the edge position of the corresponding atomic step. Reprinted with permission from [77]. Copyright 2020 American Chemical Society.

Highly oriented pyrolytic graphite (HOPG) is another representative member of a soot particles’ family and one of the components of dust present in the atmosphere. Hence, it was chosen as a model ice nucleator in another study involving ESEM [77]. The results showed an alignment of hexagonal ice particles overlapping with the direction of atomic step edges on the surface (Figure 7). It was also observed that the growth along this direction occurred at much higher rates, leading to an elongated ice hexagon along one of the a-axes. Additional DFT calculations confirmed that the-a axis of the ice crystal tends to match with the step edge during crystal growth, which is in full accordance with the experimental data acquired in the ESEM.

Although many works focus on pristine minerals for the studies of ice formation, in real atmospheric conditions, soil-derived matter composed of a mixture of mineral and organic particles contribute to the ice nucleation process. Hence, important aspects when studying cloud formation, precipitation, and soil-derived nutrient cycling are the interactions between mineral particles, biological matter and ice [78,79]. Ice formation on soil organic matter (SOM), including decomposing vegetation [80], microbes [81], fungi [82], and organic particles [83], has already been studied for many years. However, only recently these interactions were examined with the use of direct imaging through ESEM. The relevance of these interactions in comparison with pristine, non-coated samples was the focus of an investigation involving ESEM\EDX techniques as means of visualization and chemical analysis [84]. Water uptake and ice nucleation was found to take place on mineral coatings first, in field-deployed and laboratory samples. The authors point out to soil microbes as a source of those ice nucleating organic coatings. In contrast to that, the fungal−mineral contacts did not seem to impact ice nucleation activity.

Water freezing and ice formation are important from the point of view of climate, but also affect the survival of plants. The survival of some plants at temperatures below the freezing of water is conditioned by dehydration mechanisms through extracellular ice formation. This mechanism was visualized by ESEM on vesicular plants, such as *Conocephalum salebrosum* and *Marchanthia polymorpha* L. subsp. *ruderalis* in 2021 [85]. Ice formation was observed in the range of temperatures between −5 and −10 °C within the air chambers of both species, with ice crystals growing out of the air chamber pores. Additionally, a random ice crystal formation on various sites on the ventral side was also detected.

### 2.3. Ice Morphology and Dynamics of Ice Formation and Sublimation

In the initial modern studies on water freezing, around 1930s, important discoveries on ice formation were claimed based on observation by cameras and optical microscopes. The old quote that claimed that there are no two identical snowflakes [86] was confirmed by Nakaya, who classified and organized them in a “snow crystal morphology diagram” [87] based on the growth patterns of snow crystals at different temperatures and supersaturation conditions. Even though important aspects of ice formation, such as the influence of temperature and humidity, mass and heat transfer, surface physicochemical properties, have been studied ever since, the understanding of the mechanisms governing ice morphology and growth are still not fully understood. However, even though it is impossible to find two identical snowflakes in nature, Libbrecht has proven that, by carefully tuning the ice growth conditions in the laboratory, it is possible to grow two identical-twin snowflakes. In 2019, he even proposed a quantitative physical model of the “snow crystal morphology diagram”, a semi-empirical molecular model of surface attachment kinetics for ice crystal growth from water vapor. In his model, the ice crystal growth is governed by a surface-diffusion behavior sensitive to facet width and surface pre-melting [88].

**Figure 8:**
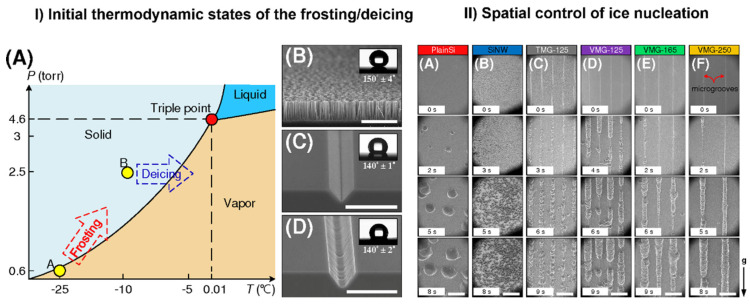
Control of ice nucleation. (**I**): (**A**) The initial thermodynamic states for frosting and deicing processes. Points A and B represent initial states of frosting and defrosting processes, respectively. SEM images of (**B**) the SiNW surface, (**C**) the VMG surface, and (**D**) the TMG surface. The inserts in (**B**–**D**) are the contact angles on the SiNW, VMG-125, and TMG-125 surfaces. Scale bars in the figure are 15 µm. (**II**): ESEM images of ice nucleation on (**A**) plain silicon, (**B**) SiNW surface, (**C**) TMG surface with a groove spacing of 125 µm, and VMG surfaces with groove spacings of (**D**) 125 µm, (**E**) 165 µm, and (**F**) 250 µm. Scale bars in the figure are 200 µm. Adapted with permission from [111]. Copyright 2017 American Chemical Society.

With the advancement in resolution capabilities of the ESEM technique, high magnification images of ice crystals were possible to be obtained. In a study that compares hexagonal ice crystals grown in situ in the ESEM with ice crystals grown ex situ in a diffusion chamber [89], the mesoscopic topography of the crystals was revealed, showing the presence of linear striations, ridges, islands, steps, peaks, pits, and crevasses. Microscale topography was ubiquitously present at the temperatures of −10 to −40 °C on all crystal facets, irrespective of the substrate under subsaturated and supersaturated conditions (Figure 8). However, the roughness patterns on the sublimated and transferred crystals were found to be drastically different, most probably due to the different growth conditions.

Very recently, the complete process of ice growth and sublimation on surfaces was followed by in situ ESEM, from the initial ice growth through the coalescence of the crystals and formation of polycrystalline film to the sublimation of ice on an oxidized Si wafer [90]. The final surface of ice, after the merging of individual crystallites into a polycrystalline film, was dominated by grain boundaries and defects (pores), which were pointed out as areas where the sublimation of ice originates. These pores were most probably created by multiple grain boundaries, as stated by the authors. We believe it is important to note that the authors took special care in avoiding the beam damage by using low magnification and short exposures in their ESEM experiments. Moreover, they call for a further development of the ESEM capabilities to come closer to atmospheric conditions, such as being able to introduce more gases simultaneously or improve the sensibility of the detection system. It is true that the pure water conditions found in ESEM are far away from the realistic conditions of the troposphere, where a mixture of gases and particulate matter exist. However, recent computer simulations point out that nitrogen, the main component of the atmosphere, has very low adsorption energy on ice. However, it seems that the density of nitrogen found in the atmosphere may slow down the water molecules directed to the surface of ice [11]. It seems worthwhile to note that the first detailed visualization of the formation of an ice grain boundary in the ESEM was claimed in 2011 [91]. Based on that investigation, an increased probability of molecular surface disorder in the vicinity of a grain boundary was already suggested. The authors observed a transition of a facet structure of an ice hexagon from smooth to wavy when merging two adjacent hexagonal ice crystals. Prior to the above-mentioned studies, the appearance of trans-prismatic strands, separated from one another by distances of 5–10 µm on the hexagonal ice crystals during cycles of growth and ablation, especially in near the frost point conditions, was reported [92]. The investigation in this case was undertaken inside a variable pressure SEM (VP-SEM) where the water vapor was created from a metal reservoir placed inside the chamber. Once again, mainly due to the low-pressure conditions of VP-SEM, it was argued that such observed mesoscopic structures may also exist in cirrus ice under certain conditions. An extensive study showed that abnormal behaviors of the gas–liquid–solid phase transitions of water were found at varying pressures in the ESEM [93]. The formation of supercooled water droplets prior to ice formation was found at a pressure of 550 Pa and temperature of −7 °C. This and other unexpected results of this work shone a new light for the better understanding of the water phase diagram. Other authors defined three steps in the sublimation of ice to explain the morphology development during this process: initial random desorption, kink formation along the prismatic planes, and finally the subsequent ridge formation due to the coalescence of these kinks [94].

During the process of water freezing, the impurities present in it are trapped inside to become a part of the structure of the formed ice and/or snow. These impurities are stored, transformed, and eventually released from the crystallized water. The information about the location and speciation of this contamination under varying environmental conditions is critical for assessing their reactivity and fate [95,96,97]. The most important impurities in polar regions are ions originating from sea salt, such as Na^+^, Cl^−^, and Br^−^, and have been observed in snow, ice cores, and aerosols forming a large chemical reservoir [98,99,100,101]. The evaporation process of frost flowers created from a NaCl solution by ESEM was investigated with temperatures reaching down to −30 °C and pressures up to 2000 Pa [102]. Answers to previously unexplained observations, such as frost flowers not being a direct source of sea salt aerosols and that saline ice crystals under evaporation could accelerate the heterogeneous reactions of bromine liberation, were provided. In another study, authors used two types of detectors, the secondary electrons detector and the backscattered electrons detector combined with fluorescence spectroscopy, to obtain a unique information set about the morphology and composition of ice grain boundaries and contamination trapped inside the ice [103]. The brine layer over the ice grains was observed along with the uranyl salt trapped in the grain boundaries. The uranyl salt solution was used to visualize where the contamination ends up after the ice formation. It was found that the uranyl salt has a tendency to form channels between the ice grain boundaries. However, it was not always true, because the uranyl ion speciation was largely dependent on experimental conditions. These kinds of experiments could help to fine tune the conditions to produce cleaner and better quality ice for the food industry, through the elimination of impurities, but much more studies are needed that take into account the physical and chemical properties of specific contaminants.

From an industrial point of view, ice formation and accretion can present a serious problem causing severe accidents in certain areas, such as transportation, power networks, infrastructures, aviation, wind turbines, among others. Hence its prevention is of high importance [104,105,106]. In the last decade, superhydrophobic (SHB) surfaces have been proposed as anti-icing and deicing solutions [107,108,109,110]. However, no surface is able to completely avoid frost formation at very cold temperatures. The spatial control of ice growth and confinement of ice-stacking direction was demonstrated on a v-shaped and trapezoid-shaped microgroove patterned surface [111]. The performance of the surface was compared with plain Si surface and Si nanowire (SiNW) array-coated surface under ESEM (Figure 8). The control and confinement were accomplished by controlling local free energy barrier for frosting. Very recently, it was shown that a metal/polymer composite may also form an icephobic surface and thus mitigate the ice accretion [112]. The nickel-based skeleton provides a robust durable framework while polydimethylsiloxane (PDMS) adds the icephobicity to the compound. The authors used ESEM to obtain an insight on ice growth and its diminishment on the Ni foam/PDMS layer, confirming the good icephobicity of the two-phase structure.

**Figure 9:**
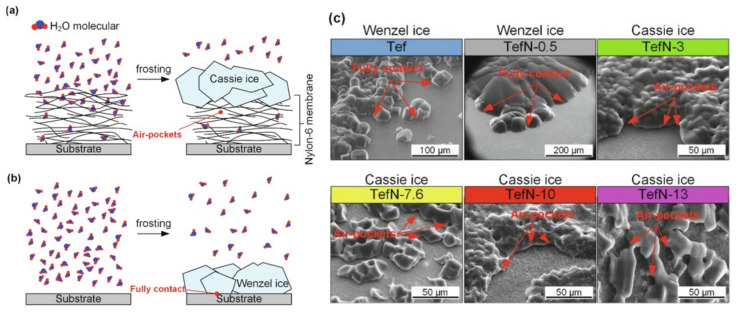
Ice morphology on studied surfaces. (**a**) Cassie ice yielded by a layer-by-layer stacked structure. (**b**) Wenzel ice formed on the surface without a membrane coating and on the surface with a thin layer of membrane. (**c**) Ice morphologies on surfaces: Wenzel ice on the Tef and TefN-0.5 surfaces and Cassie ice on the TefN-3, Tef-7.6, TefN-10, and TefN-13 surfaces. Adapted with permission from [115]. Copyright 2021 Elsevier.

Using SHB surfaces is not the only approach for anti-icing. Another approach consists of creating hydrophilic polymer coatings [113,114], which are often highly hygroscopic. The process of absorbing water molecules from the air delays ice formation. The ease of deicing of surfaces was investigated on the nylon-6 nanofiber membranes and compared with non-coated surfaces. The frosting and defrosting studies were conducted in the ESEM chamber to visualize the process of formation of either the loosely attached Cassie ice on the membrane-coated surface (the freezing occurred when water was in a Cassie state with air trapped between the surface and the ice) or Wenzel ice in full contact with the uncoated surface (liquid water collapsed into the roughness grooves to reach a more stable configuration before ice formation) [115] (Figure 9).

As previously mentioned, the electron beam interaction with the specimen under observation may play a role in some, if not all the studies conducted under ESEM. However, even if counter-intuitive, this effect may be used to the advantage of a study. The most recent example is the study of the growth of amorphous solid water (ASW) on the electrically charged surface of sapphire, used as a source to charge the electrons coming out from the gun of the scanning electron microscope [116]. After the charging of the selected area in a high vacuum mode at a low temperature, the authors switch to the low vacuum mode with water vapor as a gas environment. This strategy allows for the formation of ASW pillars on sapphire, the polymorph also found on interstellar dust, in dense molecular clouds, on comets, and planet satellites.

## 3. Conclusions

The playwright and theatre director Henrik Ibsen first said, “A thousand words leave not the same deep impression as does a single deed”. After his death in 1906, this quote was rephrased into what we know now, “A picture is worth a thousand words”, which is now a common saying. This explains very well the value that the ESEM technique has in the field of the understanding of heterogeneous ice nucleation. It has helped to visualize and to better understand the processes governing water transitions from gas, to liquid, to solid, and vice versa. The ice nucleation, growth and sublimation on particles, surfaces and organic matter were visualized in the ESEM and characterized spectroscopically by many scientists providing relevant information on the subject. We believe one of the main advantages of ESEM is the ability to determine with nanometric resolution the sites where ice nucleation takes place and even visualize the initial steps of ice nucleation, with crystals in the order of only hundreds of nm. This lateral resolution combined with the possibility of chemical analysis of the surface with the same resolution opens a wide field to investigate the main question about ice nucleation still with more shadows than light; what makes a site on a surface or particle so special to induce ice nucleation more efficiently than another sites. So far, the experiments shown in this paper mainly explored ice nucleation on “natural” materials, such as feldspars, silica, graphite, and plants. In this paper, by “natural” we mean materials not designed with specific properties or functionalities. However, few examples of designed surfaces have shown some interesting properties towards water and ice, such as the hydrophilic islands on superhydrophobic surface. We believe with the capabilities of nanotechnology and ESEM resolution, more of these kinds of special surfaces can be designed and fabricated with sites of well-defined properties as models to determine how different surface properties interplay to induce ice nucleation. The same can be applied for surfaces designed to control ice dynamics spatially, as observed in Figure 6. An important drawback of the technique is the time resolution, i.e., the frame rate especially if compared with high-speed optical microscopy. This is too slow to be able to follow initial ice nucleation or the complete history of the evolution of the ice/air interfaces. Even though this technique was developed more than three decades ago, it is still evolving and hopefully the instrumentation and software advances in this field will allow us to recreate in a more accurate manner the conditions found in the troposphere. With the development of the ESEM detectors, the resolution will also improve and enable to visualize the processes of water transformations in more detail.
